# Local Mechanical Stimuli Regulate Bone Formation and Resorption in Mice at the Tissue Level

**DOI:** 10.1371/journal.pone.0062172

**Published:** 2013-04-24

**Authors:** Friederike A. Schulte, Davide Ruffoni, Floor M. Lambers, David Christen, Duncan J. Webster, Gisela Kuhn, Ralph Müller

**Affiliations:** Institute for Biomechanics, ETH Zurich, Zurich, Switzerland; Friedrich-Schiller-University Jena, Germany

## Abstract

Bone is able to react to changing mechanical demands by adapting its internal microstructure through bone forming and resorbing cells. This process is called bone modeling and remodeling. It is evident that changes in mechanical demands at the organ level must be interpreted at the tissue level where bone (re)modeling takes place. Although assumed for a long time, the relationship between the locations of bone formation and resorption and the local mechanical environment is still under debate. The lack of suitable imaging modalities for measuring bone formation and resorption *in vivo* has made it difficult to assess the mechanoregulation of bone three-dimensionally by experiment. Using *in vivo* micro-computed tomography and high resolution finite element analysis in living mice, we show that bone formation most likely occurs at sites of high local mechanical strain (p<0.0001) and resorption at sites of low local mechanical strain (p<0.0001). Furthermore, the probability of bone resorption decreases exponentially with increasing mechanical stimulus (R^2^ = 0.99) whereas the probability of bone formation follows an exponential growth function to a maximum value (R^2^ = 0.99). Moreover, resorption is more strictly controlled than formation in loaded animals, and ovariectomy increases the amount of non-targeted resorption. Our experimental assessment of mechanoregulation at the tissue level does not show any evidence of a lazy zone and suggests that around 80% of all (re)modeling can be linked to the mechanical micro-environment. These findings disclose how mechanical stimuli at the tissue level contribute to the regulation of bone adaptation at the organ level.

## Introduction

The shape, structure and material properties of living organs vary according to the function they fulfill in the organism [1]. One of the major tasks of the skeleton is load-bearing and it is known that external loads are able to change bone mass and architecture [2]–[5]. Functional bone adaptation to changes in the mechanical environment has been assumed for more than a century [6], [7]. Since then, various mechanobiological experiments have been performed based on the concept of introducing controlled variations (either an increase or a decrease) in the mechanical loading and then measuring the corresponding bone response. Such studies have clearly indicated that bone mass and trabecular bone architecture are controlled by mechanical cues [3], [8]–[14]. Moreover, trabecular bone, compared to cortical bone, has shown a higher response to changes in the loading environment since individual trabeculae have the freedom to reach an arrangement which optimizes load transfer [8], [15], [16].

The process of bone adaptation as a whole takes place locally by bone forming and resorbing cells. Bone adaptation to mechanical demands is also called bone modeling, i.e. uncoupled bone formation and resorption. Bone repair using spatially coupled bone resorption and formation has been referred to as bone remodeling. However, following the suggestion that the pathways governing both bone modeling and remodeling may be of the same origin [17], we will in the following refer to this process as bone (re)modeling.

Until recently, measurements of local bone formation were only possible with histology combined with fluorescent dyes injected into the animal. The dyes incorporate into bone matrix that is newly formed at the time of injection and the use of several labels injected at different time points allows measuring where and how much new bone is formed [18]. The disadvantage of histology is that the animal has to be sacrificed for the readout, and that the assessment can only be completed in two-dimensional slices. Furthermore, labels are lost due to bone turnover when the time between the injection and sacrifice of the animal is too long, hence allowing only relatively short observation periods. Trabecular bone formation rates as measured from histological slices have been linked to high loads, however with moderate R^2^-values (0.13–0.42) [19]. A reason for the low correlation may be that comparisons were made on two-dimensional data, and that the data were from a cross-sectional study. A direct connection of local bone formation with high loads, or local bone resorption with low loads in the same animal, however, has been difficult to date due to the lack of suitable technologies to measure the locations of trabecular bone formation and resorption as well as to compute the local mechanical environment *in vivo* and in three dimensions.

This lack has given impetus to the field of computer simulations in bone research. In fact, the three-dimensional functioning of local mechanoregulation in trabecular bone at the tissue level has, so far, mostly been investigated with computational models by assuming different (re)modeling theories and comparing the resulting virtual trabecular architectures with experimental data or findings from the literature [17], [20]–[23]. Such *in silico* models are certainly able to capture major aspects of the functioning of local mechanoregulation; however, current computational models are based on a number of (partly competing) assumptions. These include, for instance, whether resorption is caused by random micro-cracks or load-driven [21], whether formation depends linearly on the mechanical stimulus [21] or whether a step function (implying an activation barrier) should be used [22]. Another assumption presumes the existence of a so-called lazy zone, i.e. a range of strains in which only balanced bone formation and resorption occurs [2]. In an earlier publication, we developed a computer model for bone adaptation where the relationship between local changes in bone mass and the mechanical strain were described by two linear functions and a lazy zone [23]. Although the model predicted structural bone parameters reliably with less than 12.1% error, the simulated rates and sites of formation or resorption did not coincide well with the experiment.

In the present paper, to investigate further how formation and resorption can be linked to the mechanical environment, we correlate sites of local bone formation, resorption and quiescence determined from time-lapsed *in vivo* micro-computed tomography (micro-CT), with the local strain distribution calculated by micro-finite element (micro-FE) models. The relation between the mechanical environment and bone formation/resorption was characterized in tail vertebrae of mice that were ovariectomized or subjected to mechanical loading. We hypothesized that regions of low local strains would, independent from the treatment, lead to site-specific bone resorption and regions of high local strains to site-specific bone formation.

## Materials and Methods

### Ethics Statement

During the animal experiments, all efforts were made to minimize suffering. All experiments were carried out under anesthesia and with the approval from the veterinary authority of the canton of Zurich, license number 171/2008 (Kantonales Veterinäramt Zürich, Zurich, Switzerland).

### In vivo Experiments and Micro-computed Tomography

**Figure 1:**
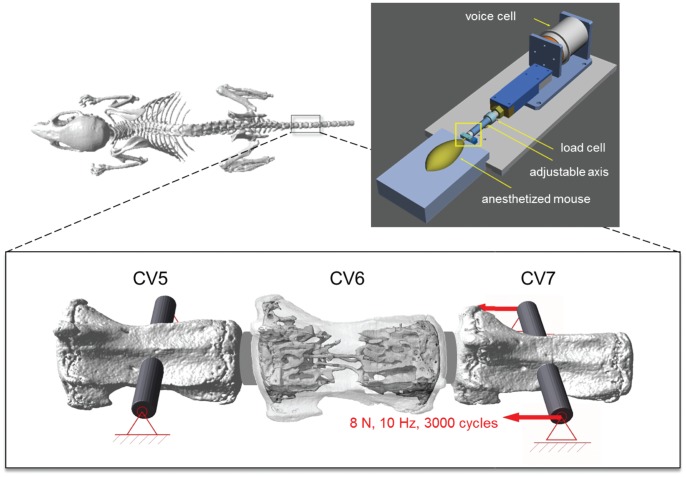
Experimental setup. The 6^th^ caudal vertebra (CV6) is cyclically loaded (10 Hz) by a force of 8 N +1 N preload. Controlled application of the force is obtained through two pins inserted in the adjacent vertebrae (CV5 fixed, CV7 displaced) through a mechanical loading device [12].

To induce a bone response under controlled loading conditions, a tail loading model [24] which permits the study of trabecular bone adaptation *in vivo* [16], [25] was used. Specifically, in 15-week-old female C57Bl/6 (B6) mice (RCC, Füllinsdorf, Switzerland), the sixth caudal vertebra (CV6) was subjected to cyclic mechanical loading at 9 N (8 N +1 N preload, CML, n = 9) through stainless steel pins inserted in the adjacent vertebrae, 3 times/week for 4 weeks at 10 Hz and 3000 cycles (Figure 1). More details about the loading regime can be found in Lambers et al. [16]. A control group (CTR, n = 8) receiving the same pins was mounted into the loading device and was given the same amount of anesthesia for a period equivalent to the loading group; however, no force was applied to the vertebra. CV6 of all animals was scanned at the start of treatment and every week for the following 4 weeks with *in vivo* micro-CT (vivaCT 40, Scanco Medical, Brüttisellen, Switzerland) at an isotropic voxel resolution of 10.5 µm. After termination of the loading experiment, a subgroup of 5 loaded and 3 non-loaded vertebrae was dissected and scanned repetitively (5 times) with repositioning between the scans [26].

A systemic, catabolic bone response was induced by ovariectomy of 15-week old female B6 mice (OVX, n = 9). A control group underwent the same surgical procedure without removal of the ovaries, which is also called sham operation (SHM, n = 7). *In vivo* micro-CT scans of CV6 were performed on the day of operation and consecutively every two weeks over a twelve-week period [27]. The reason for the larger time intervals between two consecutive scans was that the ovariectomy experiment ran over a longer period than the loading experiment but the mice should not be exposed to more radiation than necessary.

**Figure 2:**
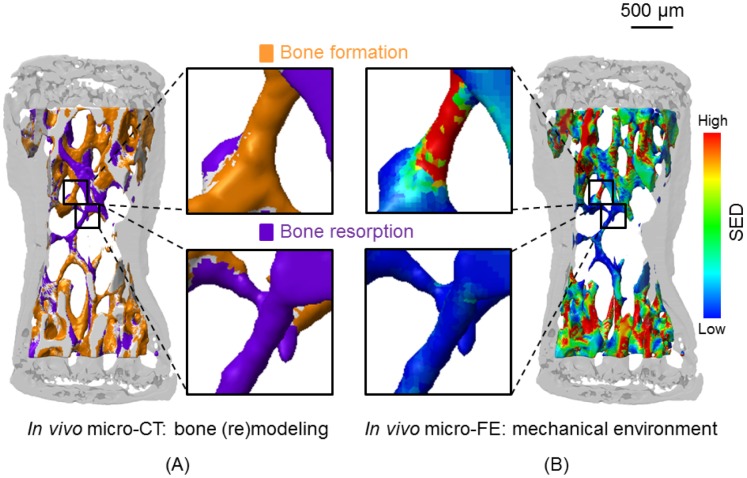
Comparison of local bone formation and resorption sites with the mechanical environment. (A) Three-dimensional trabecular bone formation and resorption sites measured with *in vivo* micro-CT over 4 weeks. The inset shows a magnified view of formation and resorption locations in individual trabeculae. (B) Corresponding SED computed with micro-FE in the basal scan. The same regions as in (A) are enlarged. A visual comparison reveals that high SED (red) matches with sites of bone formation (yellow), while low SED (blue) is found at locations of bone resorption (violet).

The time-lapsed greyscale micro-CT measurements were registered using a rigid intensity-based, least-squared registration method, allowing arbitrary rotations and translations [28]. B-Splines were chosen as the interpolation technique where a registration error of less than 1.4% was found [26]. After registration, the three-dimensional (3D) volumes were Gaussian-filtered (sigma = 1.2, support = 1) and binarized at a global threshold of 22% of the maximum greyscale value [29]. Bone mineral phantoms were scanned weekly, to ensure that the threshold was at the same mineral density for each time point. Superimposition of the binarized *in vivo* micro-CT scans of the same vertebra taken at different time points allowed the identification of bone formation and resorption sites (Figure 2A) [25]. The assessment of bone formation and resorption from *in vivo* micro-CT was proposed in Schulte et al. [25] where a comparison with histomorphometry yielded correlation coefficients R of 0.68 and 0.78 for mineral apposition rate (MAR) and mineralizing surface (MS).

### Finite Element Simulations

3D micro-FE models were generated by converting all voxels of the micro-CT image to 8 node hexahedral elements, with each model consisting of approximately 1.8 million elements (Figure 2B). A Young’s modulus of 14.8 GPa and a Poisson’s ratio of 0.3 [12] were assigned. To prevent the formation of unrealistically high strains situated on only a few nodes of the finite element mesh, loads were applied to CV6 through simulated intervertebral disks, having a circular cross sectional area of 260 µm and a height corresponding to maximum 10% of the full vertebral length. For numerical issues of the finite element solver, these disks were assigned the same Young’s modulus and Poisson’s ratio as bone. The top of the model was displaced by 1% of the total vertebral length, while the bottom was fixed. As our FE models were linear elastic, the resulting reaction force was rescaled to the value of the force applied in the experimental setup; consequently, all the FE outcomes were rescaled accordingly. Each model was solved with ParFE [30] running at the Swiss National Supercomputing Centre (CSCS, Lugano, Switzerland) with 128 CPUs in less than 60 seconds.

Strain energy density (SED), defined as the increase in energy associated with the tissue deformation per unit volume, was used as the mechanical signal. For simplicity, the time dependence of the loading was ignored because, as demonstrated by Huiskes et al. [31], (re)modeling simulations under static loading capture the main features of virtual bone evolution under dynamic loading.

The SED distribution in CV6 was calculated by applying simulated compressive loads (4 N for non-loaded [32] and 9 N for loaded mice). While applying a force of 9 N to the loaded mice is representative of the mechanical load applied in the experiment, the load magnitude for the control and ovariectomized mice requires more assumptions. Christen et al. [32] presented a technique to back-calculate prevalent loading forces (magnitudes and directions) by using the bone microstructure as input, and testing various loading scenarios with respect to the most uniform SED distribution. According to this study, the trabecular architecture of the mice which were not loaded in the loading device suggested a prevalent force in the z-direction (i.e. parallel to the long axis of the vertebra) of 4 N with negligible shear/bending forces for x- and y-directions (i.e. perpendicular to the long axis of the vertebra).

### Analysis of Local Mechanoregulation

The local mechanical environment was derived from micro-FE simulations of the baseline scan (Figure 2B). Here, and in computer models of bone (re)modeling in general, SED is used as a mathematical term to describe the (re)modeling stimulus phenomenologically. To quantify the relationship between the local SED and cellular activity on the bone surface, the bone formation or resorption sites determined by rigid registration were projected onto the surface of the baseline scan (considering a 6-neighborhood topology), resulting in three masks, representing three different clusters of formed (F), quiescent (Q) or resorbed (R) bone. Figure 2A shows a three-colored image with formation sites in yellow, quiescent sites in grey and resorption sites in violet. The mean SED value was calculated in each of these three masks and for each mouse, and as absolute SED values differ per mouse and over time due to differences in BV/TV, normalization to the mean SED of the quiescent surface was performed. The amount of formed, quiescent or resorbed voxels was calculated for each group and at each time point to gain more insight into the effect of the different treatments. Furthermore, to establish a quantitative description of the mechanoregulatory system and following the same strategy adopted to describe previous (re)modeling theories [2], [17], [22], [33], the relation between increasing mechanical stimuli and consequent (re)modeling events was investigated. Experimental strain-related (re)modeling rules for the behavior of osteoblasts and osteoclasts as a function of the local mechanical stimulus were obtained by analyzing and comparing the frequency distributions of SED in the three clusters of formed, resorbed and quiescent bone. Thus, for each value of SED (binned at 1% step size of the maximum SED), the relative percentage of voxels being formed, quiescent and resorbed can be interpreted as the probability for a given (re)modeling event to occur. Furthermore, all SED values in this analysis were normalized by the maximum value observed in each animal to allow for a comparison among individual animals and treatments. Additionally, before computing the (re)modeling rules, it was assumed that each (re)modeling event has the same occurrence probability (i.e., formation, resorption and quiescent regions were virtually rescaled to have the same amount of voxels) to rule out the dependence on the imbalance between bone formation and bone resorption which may be due to bone growth, loading or OVX. Therefore, in mathematical terms, the (re)modeling rules are equivalent to the conditional probability of a (re)modeling event taking place within a given time interval. The (re)modeling probabilities were fitted by exponential functions using non-linear regression analysis.

As mechanoadaptation curves have typically been represented on a two-dimensional graph showing on the horizontal axis the mechanical stimulus and on the vertical axis the net bone response (i.e. net effect of (re)modeling), our data were furthermore converted into this format by subtracting the probability of resorption from the probability of formation at every binned SED value.

To ensure the effects seen were true and not due to measurement error, the reproducibility of formed/quiescent/resorbed surfaces was determined. To this purpose, the repeated *ex vivo* scans of the reproducibility study were superimposed onto the first scan repetition and the amount of erroneous formation/resorption voxels per bone volume or bone surface was calculated.

### Statistics

A two-tailed paired (within animal) or unpaired (between groups) Student’s t-test with Bonferroni-correction for multiple comparisons was performed after testing for equal variance of sample by the Kolmogorov-Smirnof test. Over time, two-tailed repeated measures ANOVA with Bonferroni-correction for multiple comparisons was used. All statistical tests were performed with R (R, Auckland, New Zealand, [34]). p<0.05 was considered significant.

## Results

**Figure 3:**
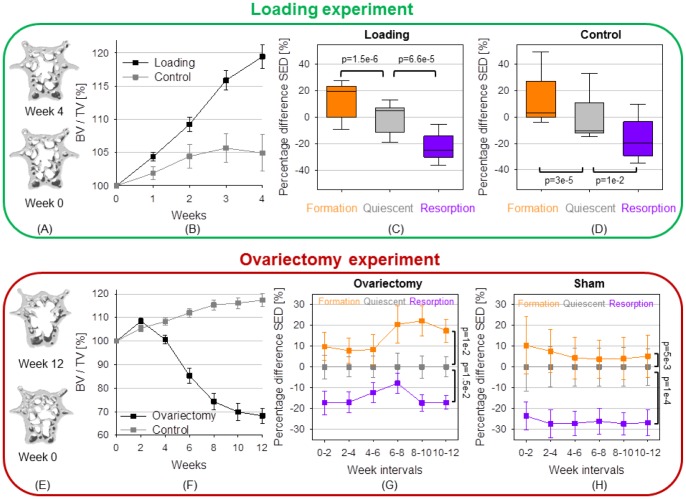
Evidence of local mechanical control for bone formation and resorption. (A) Sagittal sections through the diaphyseal CV6 at (bottom) the basal scan and (top) after 4 weeks of mechanical loading. (B) Percentage increase in trabecular BV/TV in the loading and control group. (C) Mean SED at formation, quiescent and resorption sites expressed as percent difference from the mean (over all animals) quiescent SED within the first time interval. Data are represented by boxplots, i.e. the inner box contains 50% of all data, the whisker bars denote the full range and the black line represents the median value (over all animals). (D) Mechanical regulation in the CTR group. (E) Sagittal sections through CV6 at (bottom) 0 and (top) 12 weeks after ovariectomy (OVX) (F) OVX causes a strong decrease in BV/TV compared to SHAM-operation (SHM). (G) Evidence of the mechanical regulation of the (re)modeling process in OVX. (H) Evidence of the mechanical regulation of the (re)modeling process in SHM. All data points in (F) to (H) are presented as mean ± standard error.

Cyclic mechanical loading (CML, n = 9) of CV6 over four weeks of experiment led to a significant increase in the trabecular bone volume fraction (BV/TV) compared to CTR (n = 8) and over time (19.5%, repeated measures ANOVA, p<0.05, Figure 3A, B). From week 0 to 1, mean SED at formation sites was 13.7±4.5% higher than mean SED at quiescent sites and, at the same time, mean SED at resorption sites was 22.8±3.4% lower than mean SED at quiescent sites (n = 9, Students t-test with Bonferroni-correction, both p<0.0001, Figure 3C). Table 1 contains the mean SED values in each cluster and all animal groups. In the remaining week intervals, a similar pattern was found, i.e. mean SED at formation sites was between 11.7%–16.1% higher (p<0.0001) and at resorption sites between 23.8–26.4% lower (p<0.0001) than mean SED at quiescent sites. The absolute values of SED in each cluster decreased slightly over time. This can be explained by the increasing BV/TV of the loaded animals. The percent difference to the mean SED of quiescence, however, is in the same range over all week intervals. These findings indicate that both osteoblastic and osteoclastic activities are controlled by local mechanical stimuli.

**Table 1:** Mean and standard deviation for absolute SED in formed (F), quiescent (Q) and resorbed (R) bone surfaces in all control and treatment groups.

Group	Week	F [kPa]	Q [kPa]	R [kPa]	|F–Q| in %	|R–Q| in %	p-value(F vs. Q)	p-value(R vs. Q)
CML	0–1	7.52±0.90	6.62±0.73	5.11±0.67	**13.67**	**−22.83**	1.5e-6	6.6e-5
	1–2	6.93±0.53	6.09±0.56	4.64±0.64	**13.81**	**−23.76**	5.4e-5	6.1e-7
	2–3	6.46±0.64	5.57±0.55	4.10±0.48	**16.12**	**−26.41**	2.6e-6	2.7e-7
	3–4	5.81±0.50	5.20±0.49	3.90±0.47	**11.70**	**−25.00**	4.1e-7	4.5e-7
CTR	0–1	1.60±0.30	1.40±0.27	1.17±0.24	**13.87**	**−16.41**	3.0e-5	0.00550
	1–2	1.55±0.31	1.37±0.26	1.11±0.22	**12.64**	**−19.43**	0.00090	0.00037
	2–3	1.47±0.30	1.31±0.27	0.99±0.20	**12.00**	**−24.29**	6.4e-5	0.00058
	3–4	1.41±0.30	1.27±0.25	0.97±0.19	**10.96**	**−23.81**	0.00161	0.00042
OVX	0–2	1.10±0.20	1.00±0.17	0.83±0.17	**9.77**	**−17.24**	0.00021	0.00307
	2–4	1.06±0.18	0.99±0.14	0.82±0.15	**7.85**	**−16.95**	0.010	6.1e-06
	4–6	1.22±0.24	1.13±0.18	0.99±0.17	**8.36**	**−12.29**	0.01979	0.00015
	6–8	1.69±0.37	1.40±0.27	1.29±0.20	**20.48**	**−7.88**	0.00023	0.02351
	8–10	2.00±0.36	1.64±0.26	1.35±0.19	**22.15**	**−17.29**	0.00015	0.00227
	10–12	1.96±0.28	1.67±0.24	1.38±0.16	**17.46**	**−17.08**	6.1e-05	0.0029
SHM	0–2	1.33±0.44	1.21±0.37	0.92±0.21	**10.34**	**−23.57**	0.013	0.014
	2–4	1.25±0.32	1.16±0.30	0.85±0.21	**7.58**	**−27.35**	0.00041	0.00057
	4–6	1.18±0.30	1.13±0.27	0.83±0.17	**4.34**	**−27.13**	n.s.	0.0012
	6–8	1.14±0.26	1.10±0.27	0.82±0.18	**3.74**	**−26.07**	0.01804	0.00095
	8–10	1.14±0.29	1.10±0.27	0.80±1.53	**3.98**	**−27.32**	n.s.	0.0016
	10–12	1.13±0.29	1.07±0.25	0.79±0.18	**5.13**	**−26.76**	0.04208	0.00032

Furthermore, the average difference from the mean SED in quiescent surfaces is given in %, as well as the p-values between F vs. Q and R vs. Q.

An analogous pattern with respect to mechanoregulation was observed in the animals of the CTR group (n = 8, repeated measures ANOVA with Bonferroni-correction, both p<0.05, Figure 3D, Table 1). Again, the absolute values of mean SED reported in Table 1 differed as with respect to the loaded animals, since a loading force of 4 N instead of 9 N was assumed. The fact that the same pattern was found not only for loaded but also for control mice, indicates that the same mechanism which allows bone to adapt to strong changes in the loading conditions also seems to control bone (re)modeling in daily activities.

Next, we investigated to what extent ovariectomy interferes with the local regulatory mechanism of bone (re)modeling. Following ovariectomy (OVX, n = 9), a significant loss of BV/TV caused by estrogen deficiency was measured after 12 weeks in the CV6 compared to the sham (SHM, n = 7) group and over time (31.7%; repeated measures ANOVA; p<0.0001; Figure 3E, F).

BV/TV in the SHM group, shown in Figure 3F, increased continuously and at week 4 was slightly higher than the percentage increase in BV/TV of the CTR group (Figure 3B). The absolute value of BV/TV did not differ significantly between CTR and SHM at 4 weeks. A reason for the small difference in BV/TV at week 4 may be that mice in the CTR group were anesthetized three times a week and scanned every week whereas the SHM operated animals were only anesthetized once during the operation and every two weeks afterwards for the measurements. BV/TV in SHM mice increased 16% after 12 weeks (Figure 3F). The increase in BV/TV in SHM mice is in line with the fact of continued growth in mice throughout their lifetime, with a stagnation in fast growth reported in the literature from 3 months (third lumbar vertebra of C57Bl/6 J mice) [35] to 20 months (seventh caudal vertebra in BALB/C mice) [36].

For OVX, mean SED values in the regions of bone formation (resorption) were significantly above (below) values at quiescent bone surfaces over all 2-week-time intervals (n = 9, repeated measures ANOVA with Bonferroni-correction, both p<0.05, Figure 3G). The same behavior characterized the SHM group (n = 7, repeated measures ANOVA with Bonferroni-correction; p<0.01 and p<0.001, Figure 3H; Table 1). It is worth noticing that at week interval 4–6, the percent difference between quiescence and formation increased (Figure 3G) meaning that formation occurs in an even more targeted fashion at times of high bone loss. At the same time, the difference between resorption and quiescence became smaller, indicating that high bone loss occurred through less targeted bone resorption. When bone mass stabilized after 10 weeks, the differences in mean SED returned to their original levels (Fig. 3G, week 10–12 is similar to week 0–2). Comparing Figure 3G and 3H, the resorption in SHM mice occurred at lower SED values (percent difference at approximately **−**25%) than in ovariectomy (approximately **−**18%). On the other hand, formation in OVX seemed to be more targeted than in SHM (rising up to 21% in OVX, compared to less than 10% in SHM). Taken together, our results suggest that the local mechanical regulation mechanism is still active in the case of estrogen deficiency but less targeted during times of high bone loss.

**Figure 4:**
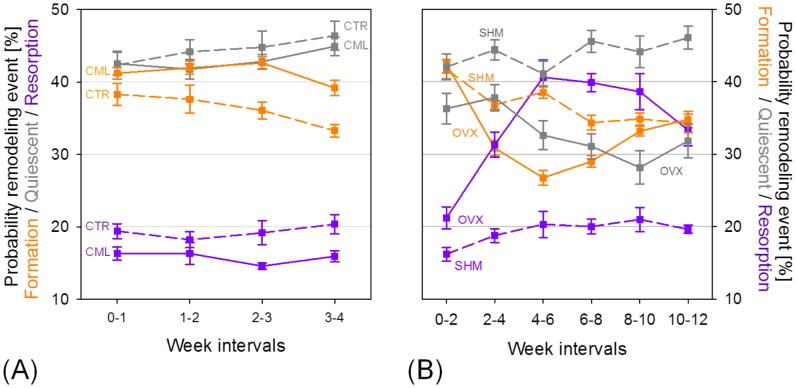
Absolute (re)modeling probabilities. (A) Mechanical loading experiment: The probability for bone formation on the bone surface is higher than for bone resorption in both the CML and the CTR group, with the effects in the CML group being more pronounced than in the control group. (B) Ovariectomy experiment: The probability of bone resorption on the bone surface of OVX increases in the first four weeks after ovariectomy, with decreasing probability for bone formation at the same time. After 4 weeks, the effects are reverted.

Figure 4 shows the absolute probability for a (re)modeling event to occur at the bone surface, computed by counting the relative amounts of voxels in the formation, quiescent and resorption clusters. In both the loading and control group, more formed surfaces than resorbed surfaces were found. Compared to CTR, CML showed an increased bone mass by increasing the probability of bone formation and decreasing the probability of bone resorption (p<0.001, Figure 4A), indicating that a larger part of the surface was occupied by formation, and a smaller part by resorption sites. Figure 4A also points out that after four weeks of mechanical loading, bone adaptation has not been fully accomplished. However, it is known that the probability of formation and resorption will look similar between CML and CTR once the loaded bone is fully adapted to the new loading conditions, as indicated by recent data on formation and resorption surfaces [37]. In the ovariectomy group, the probability of bone resorption peaked at the third week interval whereas in the same time interval the probability of formation reached a minimum, indicating that more resorbed than formed bone surfaces can be found.

**Figure 5:**
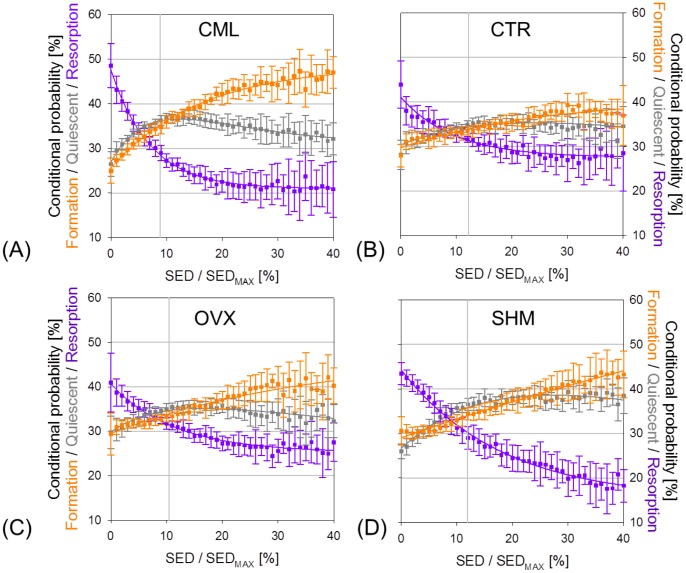
Conditional (re)modeling probabilities connecting the mechanical environment (SED) with the (re)modeling events. The mechanical regulation of bone (re)modeling is characterized by probability functions describing the so-called (re)modeling rules. When computing the (re)modeling rules, it is assumed that each (re)modeling event has the same probability of occurring to rule out the dependence on the time interval or the imbalance between bone formation and bone resorption (which may be due to bone growth, loading or OVX as shown in Figure 3). The normalized SED is truncated at 40% due to the very small number of voxels above this threshold (less than 1% of the total surface voxels). The plots show the data points (mean ± standard deviation) as well as the exponential fitting functions for bone formation, resorption and quiescence in all four experimental groups: (A) Mechanical loading group, (B) control group, (C) ovariectomy group, (D) sham-operated group.

Figure 5A shows the conditional probability curves of bone formation, *P_f_*, resorption, *P_r_*, and quiescent, *P_q_*, as a function of SED. The graph was created by averaging the individual probability curves of (re)modeling of the single animals over the first week interval. At low SED (0< SED/SED_MAX_ <6%) it was more likely for bone to be resorbed, whereas in regions of higher SED (SED/SED_MAX_ >12%), the probability of bone formation became higher. Moreover, *P_f_* and *P_q_* were very close to each other for SED ≤12%; above this value, *P_f_* constantly increased whereas *P_q_* did not vary, suggesting that bone formation may be activated only above a given stimulus. The (re)modeling curves for the CTR group were similar but not as evident as the CML curve (Figure 5B). This indicates that a strong mechanical signal enhanced bone cell response and made the mechanical control more “detectable”. The (re)modeling probabilities in SHM and OVX animals, computed at the time point when OVX showed the highest bone loss (i.e. week 4–6 in Figure 3F), showed a similar pattern as the controls of the loading experiment, thus confirming the presence of mechanical control also after ovariectomy (Figure 5C and D).

The (re)modeling rules for bone formation and resorption were fitted by exponential functions (nonlinear regression analysis, R^2^ = 0.99, p<0.001, Figure 5) where the fitting parameters can be directly linked to the functioning of the mechanosensory system. The fitting functions and resulting parameters for formation and resorption in all groups can be found in Table 2.

**Table 2:** Coefficients of the (re)modeling curves for formation, resorption and the net bone response.

Coefficient	CML (week 0–1)	CTR (week 0–1)	OVX (week 4–6)	SHM (week 4–6)
Formation: F = y0+a*(1**−**exp(**−**b*SED/SED_MAX_))				
y0	25.567	29.735	29.462	29.068
a	22.949	10.933	17.881	62.696
b	0.062	0.040	0.0275	0.0069
**R^2^**	**0.987**	**0.912**	**0.927**	**0.985**
Resorption: R = y0+a*(exp(−b*SED/SED_MAX_))				
y0	20.973	27.551	25.231	15.154
a	26.643	13.542	15.433	29.123
b	0.142	0.104	0.087	0.055
**R^2^**	**0.992**	**0.916**	**0.975**	**0.990**
Net bone response: N = y0+a*(1−exp(−b*SED/SED_MAX_))				
y0	−21.458	−11.095	−10.796	−14.762
a	47.405	22.766	29.379	53.438
b	0.101	0.075	0.057	0.035
**R^2^**	**0.990**	**0.923**	**0.965**	**0.992**

Fitting functions and coefficients of the (re)modeling curves for formation, resorption and the net bone response in all animal groups.

The offset-parameter *y0* indicates that bone resorption and formation show a certain probability to occur over the full range of mechanical stimuli which would be a fingerprint of non-targeted (re)modeling [38]. Regarding the loaded animals, the probability for bone formation and resorption independent of mechanical stimuli (i.e. offset parameter) was 25.6% and 21.0%, respectively. The amount of non-targeted (re)modeling in the estrogen depleted condition was 29.5% (formation) and 25.2% (resorption), compared to 29.1% non-targeted formation and 15.2% non-targeted resorption in the SHM group.

**Figure 6:**
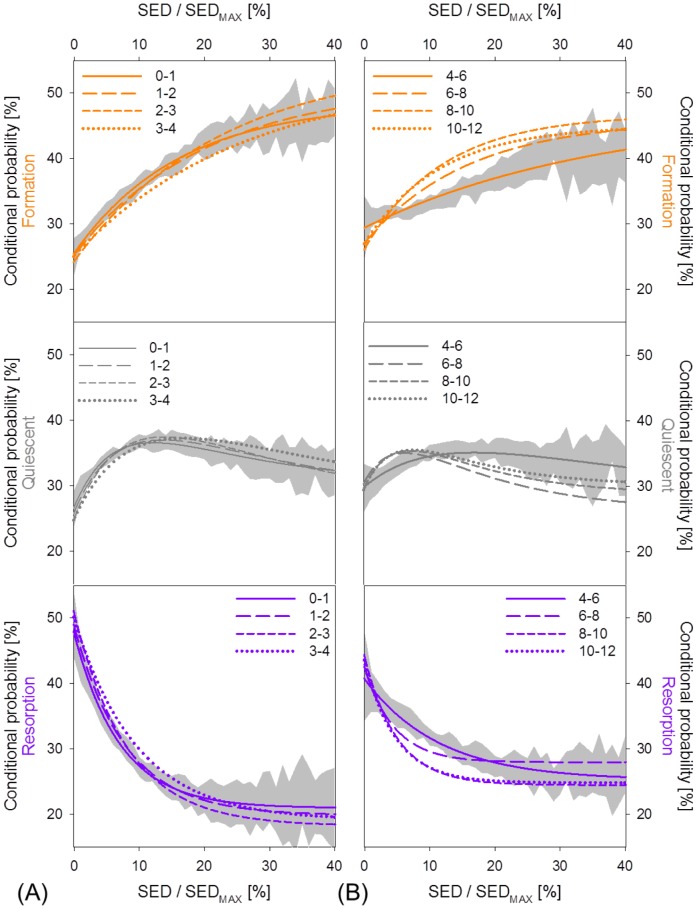
(Re)modeling probabilities of all week intervals in the CML and the OVX group. (A) For CML, the curves lie within or very close to the one standard deviation range of the first week interval, which is denoted as the grey area. (B) In the OVX group, the interval of highest bone loss (week 4–6) is characterized by an almost linear slope of bone formation. With time, this slope changes into an exponential curve. For OVX, also the curve of bone resorption becomes more exponential over time.

The parameter *a* in front of the exponent quantifies the mechanical sensitivity of the system. For OVX, a reduced mechanical sensitivity may be assumed as the extent of variation of *P_r_* and *P_f_* was approximately a factor 2 and a factor 4 smaller compared to the SHM animals. Lastly, the coefficient *b* inside the exponent can be interpreted as the amount of mechanical control in the (re)modeling process. In all animal groups, the coefficients for bone resorption were more than a factor 2 higher than those for bone formation, suggesting that the sites of bone resorption are more strictly mechanically controlled than the sites of bone formation. This means that small decreases in SED will have large effects on the resorption response while large increases in SED will only have moderate changes in the formation response. Moreover, OVX showed less mechanical control both for formation (−55%) and resorption (−39%) with respect to CML. Figure 6 shows the (re)modeling probabilities relative to all time intervals for CML and OVX. It can be seen that for mechanical loading, the (re)modeling curves lay very close together and were all located within the one standard deviation range of the first week interval (except for the last measurement, Figure 6A). In ovariectomy, the (re)modeling curves presented some variation over the different time intervals, most probably due to the acute phase of bone loss (Figure 6B).

**Figure 7:**
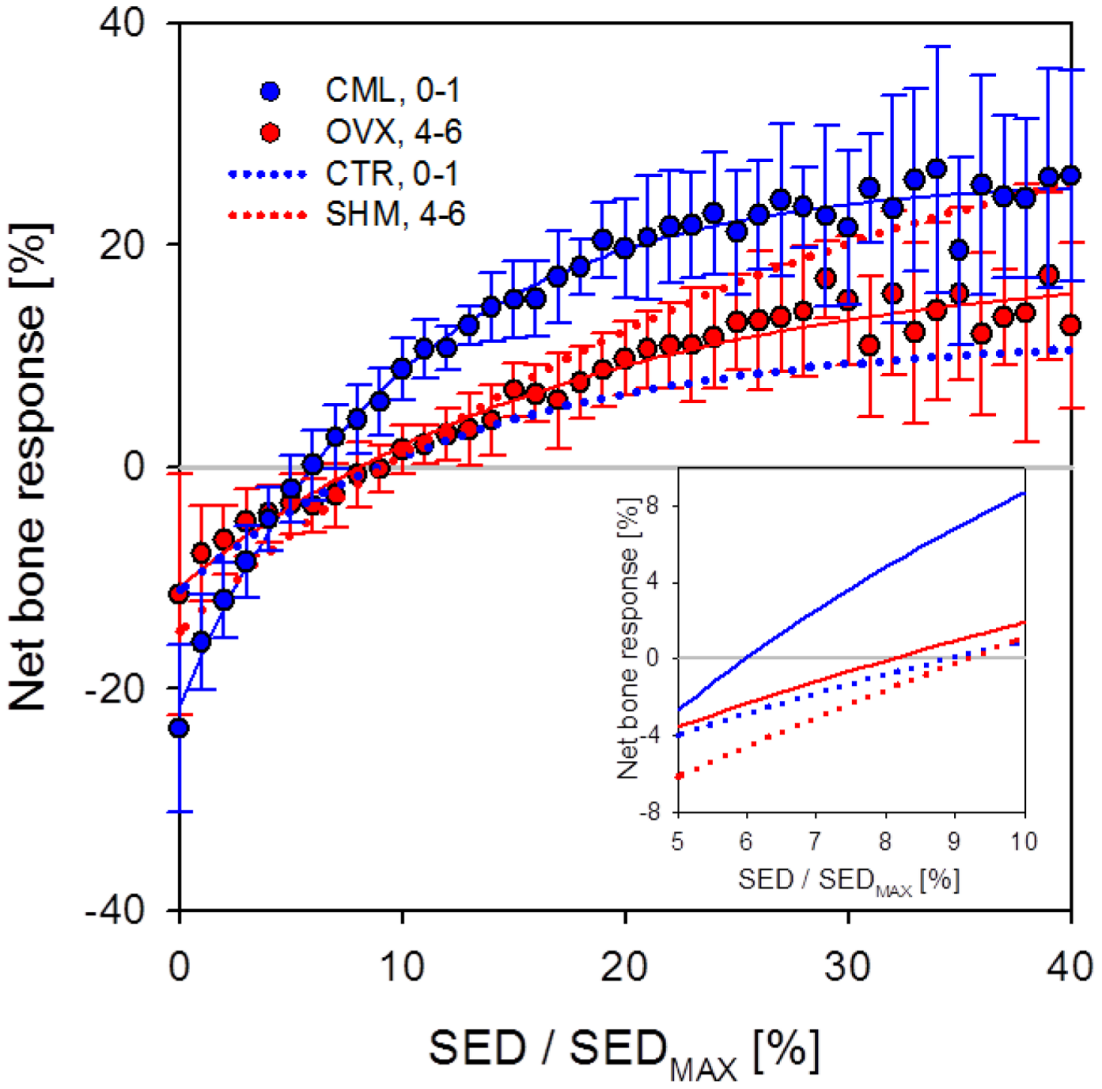
Experimental net bone response. Experimental net bone response versus normalized SED (i.e. SED/SED_max_). The curves for the CML and CTR group are shown in blue and for OVX and SHM in red. The dotted lines denote the fitting functions of the control and sham groups. It can be noted that in the CML group, the exponential behavior is more pronounced than in CTR, SHM and OVX. Also, the zero crossing point of the loading group is shifted towards the left as indicated in the magnified view in the inset. Compared to OVX, this means that in a highly controlled loading environment only lower SED values are used for bone resorption. No indication of a “lazy zone” around the x-axis can be found.

Figure 7 shows the net bone response as a function of the local mechanical stimulus. The graphs agree with the current knowledge that net bone loss occurs at low mechanical stimulus and net bone gain in the zone of high mechanical stimulus. However, a disagreement with the current understanding of bone (re)modeling is that our data give no evidence of a lazy zone. This is true for ovariectomized, loaded and control animals. Moreover, for the loading group, the relation between SED/SED_MAX_ and net bone response is well described by an exponential growth function to a maximum value (R^2^ = 0.99) whereas for CTR, SHM and OVX more linear fitting functions could also be used. Again, all exponential fitting function parameters can be found in Table 2. Furthermore, the point where CML crosses zero was shifted to the left in respect to the other groups (see inset of Figure 7) which means that only values of lower SED are leading to resorption in loaded mice. The zero-crossing-points for the CTR, SHM and OVX group were similar. For all groups, approximately the lower third of normalized SED values resulted in net bone loss, and the upper two-thirds in net bone gain, supporting the assumption that resorption is controlled more strongly than formation. The curves for CTR and SHM differ slightly in their shape. A possible reason could be that there was a time interval of two weeks for SHM, and a time interval of one week for CTR, where higher noise from erroneous voxels can be expected.

In respect to the experimental error, the amount of erroneously “formed” or “resorbed” voxels was determined from the repeated *ex vivo* measurements. The amount of erroneously formed bone volume (surface) was 5.3%±2.1% (17.8%±5.1%) and the erroneously resorbed bone volume (surface) was 4.9%±1.8% (16.5%±4.3%). It is assumed that this error mainly comes from the partial volumes effect and the registration error.

## Discussion

The objective of the current study was to investigate the relationship between the local mechanical environment and the bone (re)modeling process at the tissue level. Our results demonstrate that the loads applied globally control local bone formation and resorption at the tissue level. On top of confirming the well-known assumption stating that “bone is formed where needed and resorbed where not needed” [6], we quantified the relation between SED and bone adaptation using novel evaluation methods.

The theory of bone gain in sites of high mechanical stimuli and loss in sites of low stimuli has existed for more than 100 years [6], and the theory of local control of this process for more than 20 years [2]. Several mechanobiological experiments have been designed to connect the loading at a single skeletal location, such as the ulna or the caudal vertebra, with the subsequent changes in bone mass, as well as in the cortical and trabecular architecture [11], [12], [24], [39], [40]. For instance, cortical bone responds to sustained loading mainly by increasing periosteal bone formation at the highly strained sites [41]. This, in turn, increases the resistance to bending and torsional loading [5]. Trabecular bone also shows an adaptive response to increased mechanical loading in terms of changes in trabecular bone volume and morphometry [1], [5], [11]. For instance, Sugiyama et al. [42] and Ellman et al. [43] suggested a linear relationship between mechanical signal and bone mass changes by applying various loads (also unloading leading to bone loss) to different groups of mice. It is well accepted that unloading causes bone loss in both cortical and trabecular bone [5], [41]–[43]. Nevertheless, experimental evidence that low mechanical stimuli control bone resorption at a single site is lacking, mostly because the characterization of the spatial locations of bone resorption by histomorphometry has proved more challenging in the past than for bone formation.

The novelties of our approach are i) formation and resorption can be analyzed in 3D in a longitudinal fashion, ii) they can be analyzed separately from each other and iii) they can be linked to the mechanical signal in a single animal and at a single site. This approach allows us to determine exponential formation vs. SED and resorption vs. SED relationships with very high confidence (R^2^ = 0.91–0.99).

Our results agree with Sugiyama et al. [42] and Ellman et al. [43] that no lazy zone exists but in contrast to their suggestions, our findings favor an exponential relationship between (re)modeling and increasing SED. A reason for this disagreement might be that in previous studies it was not possible to investigate formation and resorption as two separate processes. The current data provide evidence about the local response of bone tissue to mechanical signals which is due to a coordinated action of several bone forming and resorbing cells. However, a downside of our technique may be that information on single cell behavior is still not accessible due to current limitations in image resolution.

Defining a relationship between local SED and the following bone response can also be helpful for computer simulations of bone (re)modeling. *In silico* modeling has, in the past decades, been considered a valuable tool for testing various assumptions on how the local mechanical environment can control bone formation and resorption [17], [20]–[22]. Huiskes and colleagues [17], [21], for example, proposed bone resorption to be independent of the mechanical signal and bone formation to take place linearly and only once the mechanical stimulus has exceeded a certain threshold. Adachi et al. [20], [44] simulated the net effect of bone formation and resorption by an apparent movement of the trabecular surface, driven by local stress gradients, i.e. if the local stress value is higher/lower than its direct neighborhood. Weinkamer and coworkers [22], [45] described bone formation and resorption as two separate stochastic processes and suggested the existence of an activation barrier for the mechanical stimulus above which bone formation is switched on. Though such computational studies all provided possible realistic scenarios for the regulation of trabecular bone (re)modeling in response to loading, they were not able to exclude any of the competing theories. Our experimental findings, in particular the (re)modeling rules, may be used as an input for future simulation models.

In our study, both bone forming and resorbing cell types seem to respond to the local mechanical stimulus. Here we used SED to describe the mechanical environment; however, it should be noted that SED is just one possible mathematical description of the deformation stated and must not be necessarily considered as “the” mechanical signal sensed by the cells. When converting the SED values into effective strain (which is a scalar value summarizing the strain tensor), the mean value for formation, quiescence and resorption in the loading case amounted to 1008, 945, 690 µstrain, respectively. With this, the SED values for bone formation reported here correspond to effective strains which are a bit lower than formation thresholds reported in the literature (between 1050 and 3074 µstrain) [46]–[48]. However it should be noted that it is difficult to directly compare these values with values in literature in absolute terms as different skeletal locations (e.g. tibia, femur or vertebra) are considered. Moreover, experimental measures of strain are conducted via strain gauges attached to the cortical shell (not the trabecular compartment) which typically account for only one component of the strain tensor [47], [48].

Here, we showed that both the probabilities of bone formation and resorption can be described by exponential functions of SED, with small increases in SED evoking large decreases in the probability of bone resorption. For this reason, we conclude that bone resorption is more strictly controlled than bone formation. This is mechanically sound as it is more critical when bone is resorbed at the “wrong” place than when bone is formed at the “wrong” place.

Furthermore, our results revealed a considerable portion of bone (re)modeling (15.2%–29.7%) which was not related to SED (Table 2 and Figure 4). In principle, the experimental error may influence the amount of non-targeted (re)modeling; nevertheless, assuming that the wrong voxels are uniformly distributed in each SED interval and considering the normalization that each (re)modeling event has the same probability to occur, such error mainly affects the absolute probability of a (re)modeling event taking place and not the conditional probability describing the dependence with SED (i.e. (re)modeling rules). Also, we found that with estrogen depletion, the resorptive portion not related to SED increases considerably. This finding is in line with the increasing evidence that estrogen receptors are involved in the bone cell response to strain and that the removal of estrogen may influence the availability of estrogen receptors which in turn could reduce the “accuracy” of targeted bone resorption [49]–[51]. On the other hand, this outcome also means that about 80% of all (re)modeling can be linked to mechanical demands.

In conclusion, we showed quantitatively how bone (re)modeling is regulated at the local tissue level, and how the regulation changes with different mechanical stimuli/estrogen deficiency. We believe that our findings on the mechanoregulation of trabecular bone are of major importance for a better understanding of bone diseases and the development of potential pharmacological therapies. Furthermore, we anticipate that our results close a large gap between *in silico* and experimental research of bone (re)modeling. With this insight, the research of *in silico* bone (re)modeling can further advance and develop so that better long-term predictions of bone change and the outcomes of pharmacological intervention can be gained.
